# No Influence of Transcutaneous Electrical Nerve Stimulation on Exercise-Induced Pain and 5-Km Cycling Time-Trial Performance

**DOI:** 10.3389/fphys.2017.00026

**Published:** 2017-02-07

**Authors:** Andrew W. Hibbert, François Billaut, Matthew C. Varley, Remco C. J. Polman

**Affiliations:** ^1^Institute of Sport, Exercise, and Active Living, Victoria UniversityMelbourne, VIC, Australia; ^2^College of Sport and Exercise Science, Victoria UniversityMelbourne, VIC, Australia; ^3^Department of Kinesiology, University LavalQuebec, QC, Canada; ^4^Psychology Department, Bournemouth UniversityBournemouth, UK

**Keywords:** transcutaneous electrical nerve stimulation, pacing, performance, afferent feedback, time-trial

## Abstract

**Introduction:** Afferent information from exercising muscle contributes to the sensation of exercise-induced muscle pain. Transcutaneous electrical nerve stimulation (TENS) delivers low–voltage electrical currents to the skin, inhibiting nociceptive afferent information. The use of TENS in reducing perceptions of exercise-induced pain has not yet been fully explored. This study aimed to investigate the effect of TENS on exercise-induced muscle pain, pacing strategy, and performance during a 5-km cycling time trial (TT).

**Methods:** On three separate occasions, in a single-blind, randomized, and cross-over design, 13 recreationally active participants underwent a 30-min TENS protocol, before performing a 5-km cycling TT. TENS was applied to the quadriceps prior to exercise under the following conditions; control (CONT), placebo with sham TENS application (PLAC), and an experimental condition with TENS application (TENS). Quadriceps fatigue was assessed with magnetic femoral nerve stimulation assessing changes in potentiated quadriceps twitch force at baseline, pre and post exercise. Subjective scores of exertion, affect and pain were taken every 1-km.

**Results:** During TTs, application of TENS did not influence pain perceptions (*P* = 0.68, $\eta_{\text{p}}^{2}$ = 0.03). There was no significant change in mean power (*P* = 0.16, $\eta_{\text{p}}^{2}$ = 0.16) or TT duration (*P* = 0.17, $\eta_{\text{p}}^{2}$ = 0.14), although effect sizes were large for these two variables. Changes in power output were not significant but showed moderate effect sizes at 500-m ($\eta_{\text{p}}^{2}$ = 0.10) and 750-m ($\eta_{\text{p}}^{2}$ = 0.10). Muscle recruitment as inferred by electromyography data was not significant, but showed large effect sizes at 250-m ($\eta_{\text{p}}^{2}$ = 0.16), 500-m ($\eta_{\text{p}}^{2}$ = 0.15), and 750-m ($\eta_{\text{p}}^{2}$ = 0.14). This indicates a possible effect for TENS influencing performance up to 1-km.

**Discussion:** These findings do not support the use of TENS to improve 5-km TT performance.

## Introduction

Pacing is the regulation of effort during exercise that aims to manage neuromuscular fatigue. It prevents excessive physiological harm and maximizes goal achievement (Edwards and Polman, 2013). To accomplish this, decisions are made based on information received from both internal, and external environments, to adjust exercise intensity (Edwards and Polman, 2013; Smits et al., 2014). Initially, pacing strategies are set in anticipation (Ulmer, 1996; Tucker, 2009), whilst regulation of intensity during exercise is influenced by afferent information from exercising muscle (Amann et al., 2009). Therefore, the ability to adjust or remove this information is of interest in pacing research, as there is potential to improve performance.

Exercise typically augments mechanical and chemical stimuli within the muscle, sensitizing, and activating nociceptive group III and IV afferent muscle fibers. These communicate information on actual or potential muscle damage to the central nervous system (O'Connor and Cook, 1999). Conscious awareness of this information forms the subjective sensation of exercise-induced pain (Loeser and Treede, 2008; Mauger, 2013). Pacing theory states that low signal (feedback) intensity will not trigger awareness (Swart et al., 2012). Yet, with an increasing stimulus intensity (i.e., intense exercise), conscious awareness is achieved, which results in appropriate decisions to change behavior (Swart et al., 2012; Edwards and Polman, 2013). Therefore, once nociceptive signals and consequently perceptions of pain become prominent, effort will be regulated to maintain discomfort at a tolerable level (Swart et al., 2012; Edwards and Polman, 2013; Mauger, 2014). This concept of a “sensory tolerance limit” (Gandevia, 2001; Hureau et al., 2016), likely occurs to prevent excessive physiological harm by limiting levels of fatigue (Amann et al., 2009). The sum of signals from a number of mechanisms contribute to this theory, including feedback from group III and IV muscle afferents but also feedback from respiratory muscles and corollary discharges (Hureau et al., 2016). Reductions to exercise performance and duration due to exercise-induced fatigue, are evidence of this concept (Amann et al., 2013). In addition, the importance of nociceptive information as part of a global tolerance limit has been demonstrated by altered nociceptive stimuli (i.e., induced or blocked), leading to changes in voluntary activation of muscle (Amann et al., 2009; Kennedy et al., 2014). Only a small number of studies, investigating analgesic interventions to augment afferent information have focused on exercise pacing and performance. Injecting fentanyl increases initial power output, but results in excessive fatigue (Amann et al., 2009), while ingestion of acetaminophen can increase mean power output during a time trial (TT) (Mauger et al., 2010) and during repeated-sprint exercise (Foster et al., 2014). An increased exercise intensity suggests nociceptive signals affect self-pacing. Yet, in acetaminophen studies, there was no change in pain perceptions. This suggests a threshold of perceived pain was adjusted and exercise intensity was increased to this tolerance limit. Therefore, there is merit for any analgesic intervention during self-paced exercise to adjust perceptions of exercise-induced pain allowing for an increased intensity (and possibly performance), before perceptions become prominent.

An alternative to ingested and injected analgesics is transcutaneous electrical nerve stimulation (TENS). Application of TENS involves low-voltage electrical currents administered to the skin for pain relief (Johnson et al., 2015). Analgesic effects are provided following the gate control theory of pain (Melzack and Wall, 1965), which controls transmission of nociceptive information. Specifically, stimulation provided by TENS targeting group II muscle afferent fibers excites inhibitory interneurons. This results in an attenuation of the ascending nociceptive stimuli from group III and IV afferent fibers (Sluka et al., 2013; Johnson et al., 2015). Based on this premise, it is possible that TENS could be used to attenuate nociceptive stimuli associated with aerobic exercise. Clinically, analgesic effects of TENS have been demonstrated for chronic musculoskeletal pain (Johnson and Martinson, 2007). In pain-free individuals, application can influence pressure pain thresholds (PPT). (Moran et al., 2011). During exercise in pain-free individuals, TENS application has improved tolerance by enhancing peripheral blood flow (Tomasi et al., 2015). Furthermore, when knee pain was induced, TENS has also reduced pain and restored quadriceps strength (Son et al., 2016). This suggests that TENS could be used in pain-free individuals to reduce pain perceptions, and possibly perceptions of exercise-induced pain. If demonstrated, a reduction of exercise-induced pain, in conjunction with possible alterations in muscle contractile properties and enhancement of blood flow, gives the potential for TENS application to be a performance enhancing strategy.

To the authors' knowledge, TENS has not been used in pain-free participants to modulate afferent feedback and reduce exercise-induced pain perceptions during self-paced exercise. Based on the potential to influence exercise-induced pain, the aim of this study was to investigate the efficacy of TENS administered before intense exercise to influence pain perceptions. It was hypothesized that TENS would influence the threshold for sensing exercise-induced pain, thereby increasing exercise intensity and performance for similar subjective pain perceptions. As TENS can influence muscle excitability and strength, a secondary objective was to assess within-exercise muscle recruitment (via electromyography) and neuromuscular fatigue. To allow the assessment of muscle recruitment, application of TENS was administered prior to exercise, in anticipation of approximately 30-min of post-stimulation analgesia, utilizing similar TENS settings displaying increased PPT within this period (Moran et al., 2011; Pantaleão et al., 2011). To allow for exercise to be within the post-stimulation time frame, a 5-km cycling TT was utilized, as it was anticipated this would be completed in ~10-min.

## Methods

### Participants

Thirteen recreationally active participants were recruited for this study (see Table 1). Written informed consent was provided in accordance with the Declaration of Helsinki. The inclusion of both males and females was based on indications sex has no influence on level of exercise-induced pain within the time frame proposed for this study (Dannecker et al., 2012), with repeated measures trial design minimizing any potential differences. Using a medical questionnaire, all participants were screened for risk factors including suitability to the exercise, current pain, currently taking pain medication and any prior use of TENS. Participants who reported pain (chronic or acute) or prior use of TENS were excluded from the study. Participants were asked to refrain from any physical activity causing severe fatigue in the 36 h prior, as well as any caffeine intake or pain medication 2 h prior to testing sessions. Procedures were approved by Victoria University's Human Research Ethics Committee.

**Table 1:** **Participant anthropometric data**.

	**Female *n* = 4**	**Male *n* = 9**	**Total *n* = 13**
Age (years)	27.5 ± 7.4	23.3 ± 4.2	24.6 ± 5.5
Height (cm)	166.0 ± 9.1	179.5 ± 7.0	175.3 ± 9.8
Body mass (kg)	62.2 ± 9.2	77.1 ± 7.8	72.8 ± 10.6
PPO (W)	250.3 ± 39.7	321.3 ± 23.0	299.5 ± 43.7
PPO (W/kg)	4.0 ± 0.4	4.2 ± 0.5	4.2 ± 0.5
VO_2peak_ (L.min^−1^)	2.6 ± 0.6	3.8 ± 0.3	3.4 ± 0.7
VO_2peak_(ml.min.kg^−1^)	40.9 ± 6.3	49.4 ± 5.6	46.7 ± 6.9
Pain at PPO	7.8 ± 2.9	9.0 ± 1.1	8.6 ± 1.8

*Data presented as mean ± SD. PPO, peak power output; VO_2peak_, peak oxygen consumption; Pain at PPO, rating of perceived quadriceps pain at PPO*.

### Experimental procedures

Participants reported to the laboratory for seven sessions, which included four preliminary and three experimental sessions. Prior to experimental sessions, three 5-km TT sessions were conducted to familiarize participants and ensure adequate reliability of pacing and performance (Hibbert et al., unpublished data). Furthermore, during the first session participants were also familiarized with TENS, peripheral magnetic stimulation protocols and algometry (see procedures below). The final preliminary session was a peak oxygen uptake (VO_2peak_) assessment to characterize participants' cardiorespiratory fitness. For experimental testing, on three different days separated by a minimum of 48 h, participants performed three 5-km cycling TTs in a single-blind randomized order: control (CONT), placebo with sham TENS application (PLAC), and an experimental condition with TENS application (TENS). TENS was applied for 30-min before performing a cycling TT (see TENS procedure below). Maximal voluntary contraction (MVC) force, responses to magnetic stimulation of the quadriceps and PPT were measured before TENS application (BASE), as well as pre (PRE), and immediately post (POST) exercise.

Upon reporting to the laboratory, participants were fitted with electromyography (EMG) electrodes (see below). A warm up of MVC and magnetic stimulation (see below) was conducted before BASE measurement. Participants were then fitted with TENS electrodes for 30-min of TENS application. A cycling warm-up (5-min cycling at 75 Watts) was conducted before PRE measurement of MVC. Immediately following PRE measurements, a 5-s sprint was conducted for EMG normalization purposes. Following this, the TT commenced after a verbal 3-s countdown from the researcher. To overcome flywheel inertia, participants were instructed to obtain a self-selected comfortable cadence immediately prior to beginning the TT. All exercise was conducted on a Velotron Pro cycle ergometer (RacerMate Inc., Seattle, WA, USA). Within the familiarization sessions, participants set the ergometer to their own specifications with values recorded and replicated for subsequent sessions. All TT protocols were controlled via Velotron Coaching software (Version 1.6.458, RacerMate Inc.) with all courses being flat with no wind effect. Participants were permitted to drink water *ad libitum* during trials. Participants were instructed to finish the required distance “as quickly as possible,” being free to change gear and cadence throughout the TT as desired. Participants were blinded from information except for distance covered. Upon TT completion, participants were quickly assisted in moving to the MVC and magnetic stimulation apparatus, for POST assessment.

### VO_2_ assessment

After TT familiarization sessions, to characterize participants, VO_2peak_ was assessed via a maximal incremental test. A ramp protocol was utilized, that equated to 30 Watts/min which commenced after a 3-min baseline period, cycling at 0 Watts (Vanhatalo et al., 2007). As participant familiarity with cycling varied, a similar test was chosen to that used for participants unfamiliar with cycling (Williams et al., 2012). Expired gas was collected and analyzed every 15-s (S-3A/I (O2) and CD-3A (CO2), AEI Technologies Inc., Pittsburgh, PA). Prior to each test, gases were calibrated with known concentrations and flow calibrations were performed using a 3-L calibration syringe. Participants were encouraged throughout the final stages and the test ceased when the participant could not maintain a cadence above 60 rpm or volitional fatigue was achieved. Peak oxygen uptake was calculated as the highest 30-s mean VO_2_ and peak power defined as the highest power at test conclusion. Subjective ratings for exertion (RPE) and quadriceps pain (pain scale) were measured every minute.

### Transcutaneous electrical nerve stimulation (TENS)

Participants were acutely treated with TENS (N602 ProTens; Everyway Medical Instruments, New Taipei City, Taiwan) for 30-min prior to the exercise protocol. Two TENS units were used so that the area of stimulation could be increased, with one unit (two channels) dedicated to each leg. Stimulation was applied through 50 × 90 mm adhesive electrodes (Allcare; Everyway Medical Instruments, New Taipei City, Taiwan). TENS electrode placement occurred after BASE MVC measurement, with sites shaved before placement. At the conclusion of stimulation, TENS electrodes were removed. For electrode placement, participants were asked to lay in a supine position and perform a knee extension. Two electrodes were placed on the superior portion of the quadriceps, inferior to the inguinal fold over the areas of contracted muscle bulk. Two electrodes were also placed over the inferior portion of the quadriceps, one over the vastus lateralis and one over the vastus medialis. TENS was delivered in constant mode with settings fixed at a pulse width of 200-μs, and frequency of 82.6-Hz (Chen and Johnson, 2010). The duration and settings (pulse width and frequency) of TENS was based on previous studies showing the effect of TENS on PPT (Moran et al., 2011; Pantaleão et al., 2011). In this investigation, for the assessment of muscle recruitment and practically of exercise, application of TENS was administered prior to exercise in anticipation of approximately 30-min of post-stimulation analgesia (i.e., increased PPT) as observed in previous studies (Moran et al., 2011; Pantaleão et al., 2011). Prior to study commencement, settings for the TENS units were verified using an oscilloscope (Rigol DS1054). Following calibration checks, transparent tape was placed over these controls to prevent any adjustment. For TENS application, participants were instructed to adjust intensity (via manual dials) to a level of non-painful tingling below a level that evokes involuntary muscle contraction (Moran et al., 2011; Pantaleão et al., 2011; Son et al., 2016). Throughout 30-min TENS application, participants were asked to periodically increase intensity to ensure this remained at desired level. For PLAC condition, the same electrode placement was used, but stimulation intensity was set by the researcher. To appear that stimulation was present, the TENS unit power indicator was illuminated, although the equipment did not provide stimulation. For this condition, participants were told stimulation was set to a sub-sensory level (Cheing et al., 2002). For TENS condition, participants were informed that they were receiving high TENS and low TENS for PLAC condition. In both conditions, participants were advised that they may or may not feel any stimulation and in the absence of sensation, stimulation was still active and providing analgesic effects. To account for time taken for TENS application, during CONT condition, participants laid quietly in a supine position for 30-min.

### Electromyography

Electromyographic (EMG) activity of six muscles (*vastus medialis, vastus lateralis, rectus femoris, biceps femoris, medial gastrocnemius, and gluteus maximus)* was recorded from the right lower limb via Ag/AgCl bipolar rectangular electrodes with a diameter of 30 × 20 mm and an inter-electrode distance of 20 mm (Blue Sensor N-00-S, Ambu Medicotest A/S, Ølstykke, Denmark). All signals were recorded continuously at 1500 Hz via a wireless receiver (Telemyo 2400 GT, Noraxon Inc., USA). Prior to electrode placement, the limb was shaved and abraded to minimize skin impedance, and appropriate electrode placement and functionality was checked before the start of each test. When the position of quadriceps electrodes overlapped with TENS electrode placement, electrode location was marked with a waterproof felt-tip pen to ensure reliable electrode replacement within session. All electrode sites were marked for reliable placement between subsequent testing sessions. To avoid artifacts from lower-limb movements, the electrodes were well secured with rigid tape. Raw EMG signals were band-pass filtered (12–500 Hz), were full-wave rectified and Root Mean Squared using Noraxon software (MyoResearch XP version 1.08.27). Mean RMS for individual muscles was analyzed for 20-s at 250-m intervals of TT distance. Individual muscle RMS values were summed to estimate general muscle electrical activity (RMS_sum_) (Billaut et al., 2010), and is reported as a percent of the individual maximum value obtained during a pre-exercise sprint (O'Bryan et al., 2014).

### Peripheral magnetic stimulation

Stimulation of the femoral nerve and quadriceps muscle was conducted using a magnetic stimulator (Magstim RAPID^2^; JLM Accutek Healthcare, Homebush, NSW) and a double 70-mm coil (Katayama et al., 2004; Amann et al., 2007; Billaut et al., 2013). Force responses were obtained at 1 kHz from a calibrated load cell (Extran 2kN “S” beam, model SW1, Applied Measurement, Melbourne, Australia). The load cell was connected to a non-compliant strap, which was attached around the participant's leg just superior to the malleoli of the ankle. Voluntary force and neuromuscular testing was conducted at BASE, PRE (~3-min pre-exercise) and POST (between 40 and 60-s post-exercise). Allowing for removal of TENS electrodes and application of EMG electrodes, the delay between the end of TENS application and PRE, was approximately 10-min. Time taken from the start of PRE assessment to TT start was ~6-min.

To determine the area of stimulation associated with the largest quadriceps twitch (Q_tw_), the coil head was positioned high onto the thigh, between the quadriceps muscle and the femoral triangle (Katayama et al., 2004; Amann et al., 2007; Billaut et al., 2013). This position was marked and kept the same for all trials. At BASE a warm-up was conducted with brief (~5-s) submaximal voluntary contractions increasing to a MVC separated by ~40-s. To indicate maximal depolarization of the femoral nerve, a ramp protocol of increasing stimulus intensity (from 70 to 100%) was used to achieve a plateau in BASE Q_tw_ (Katayama et al., 2004; Amann et al., 2007; Billaut et al., 2013). A near plateau was achieved in all participants at 95–100% stimulator output. For assessment, the stimulus power was set at 100% of maximum, and single stimuli were delivered. During a 5-s MVC of the quadriceps, the femoral nerve was stimulated (superimposed single stimuli) to determine the completeness of muscle activation (Katayama et al., 2004; Amann et al., 2007; Billaut et al., 2013). Stimulation was administered when the researcher visually identified a plateau in torque (Tofari et al., 2016). Three potentiated quadriceps twitches (Q_tw, pot_) were obtained 5-s after the MVC. This procedure was performed three times at BASE and PRE (60-s of rest in between) such that nine Q_tw, pot_ values were obtained, with the Q_tw, pot_ averaged and analyzed for peak force. The procedure was only performed once at end-exercise to reduce post-exercise assessment time and limit recovery as much as possible (Billaut et al., 2013). Surface EMG was used to assess the membrane excitability via muscle action potentials (M-waves) during potentiated twitches, with peak to peak duration and amplitude measured. With single stimuli delivered during the MVCs the quadriceps central activation ratio (CAR) was calculated as the percentage of voluntary force obtained during the superimposed contraction, that is, CAR = MVC ÷ (MVC + stimulated force) (Kent-Braun, 1999; Tofari et al., 2016). Stimulation was delivered on visual identification of torque plateau, and in some cases, it occurred before or after the torque plateau. To account for this, a correction equation was used where torque was averaged over 100-ms before superimposed peak (Marshall et al., 2014; Tofari et al., 2016). Due to technical problems, some CAR data were not included, and these participants have been removed from analysis with total *n* = 10.

### Perceptual scores

A 6–20 scale of rated perceived exertion (RPE) (Borg, 1970), an 11-point bipolar feeling scale (FS) (Hardy and Rejeski, 1989) and a pain scale (O'Connor and Cook, 2001) were used to assess perceived effort, affect and perceived quadriceps muscle pain. Prior to commencing the study, participants were given instructions and explained all scales. During familiarization and experimental TTs, ratings were recorded at every kilometer. At the conclusion of the study, participants were asked to subjectively access the effectiveness of TENS application. Participants were asked to rate on a 1–10 scale (1; a bit, 10; a lot), the relief from pain, and impact on performance that TENS application provided. Responses were received for both TENS and PLAC conditions.

### Algometer

Measures of PPT were recorded for the pressure applied to the quadriceps with an algometer (FPX algometer; Wagner Instruments) with a 1-cm^2^ application surface. Recordings displayed in kilograms of force (kgf) were taken from the left leg at BASE, in the last minute of TENS application (i.e., PRE exercise) and at POST. Assessment site was the midpoint between anterior superior iliac spine and head of the patella. Recordings were taken with pressure applied to relaxed muscle at a rate of 1 kg^.^cm^−2.^s^−1^. Participants verbally reported the first point when pain (distinct from pressure or discomfort) occurred, the algometry was immediately removed and corresponding measurement recorded as PPT (Moran et al., 2011).

### Statistical analysis

Conditions are defined as control (CONT), placebo with sham TENS application (PLAC), and an experimental condition with TENS application (TENS). All data was analyzed using SPSS (version 22, SPSS Inc., Chicago, IL.), with data are reported as mean ± SD. Tests for homogeneity of variances were performed to ensure normality of the cohort for dependent variables. With normality confirmed, two-way repeated measures ANOVAs (Condition × distance) were used to analyze changes in RPE, FS and pain. One-way repeated measures ANOVAs were used to analyze changes in total duration and average power, as well as 1-km duration and mean power. Two-way repeated measures ANOVAs (Condition × time) were used to analyze changes between BASE, PRE, and POST measurements for MVC, evoked response to magnetic stimulation and PPT. Percentage change between measurements (BASE-PRE and PRE-POST) was also investigated. To investigate pacing profiles, mean power and RMS_sum_ were analyzed over 250-m using repeated measures ANOVAs (Condition × distance). Given inter-participant differences in TTs, power output is expressed as a product of an individual's mass (W/kg). When sphericity was violated, Greenhouse-Geisser correction was used to adjust degrees of freedom. Paired samples *t*-tests were used to analyze subjective ratings of the TENS. For a significant main effect, *post-hoc* comparisons were examined with a one-way repeated measures ANOVA with Sidak multiple comparisons and paired sample *t*-tests. Statistical significance levels for all tests was set at *P* < 0.05. Effect sizes for ANOVA are reported as partial eta squared ($\eta_{\text{p}}^{2}$) with a small effect at 0.01, medium effect 0.06 and a large effect > 0.14. Effect sizes for *t*-tests are reported as Cohen's *d* with a small effect at 0.2, medium 0.5, and large >0.8 (Cohen, 1988).

## Results

### Perceptual scores and pain

**Figure 1:**
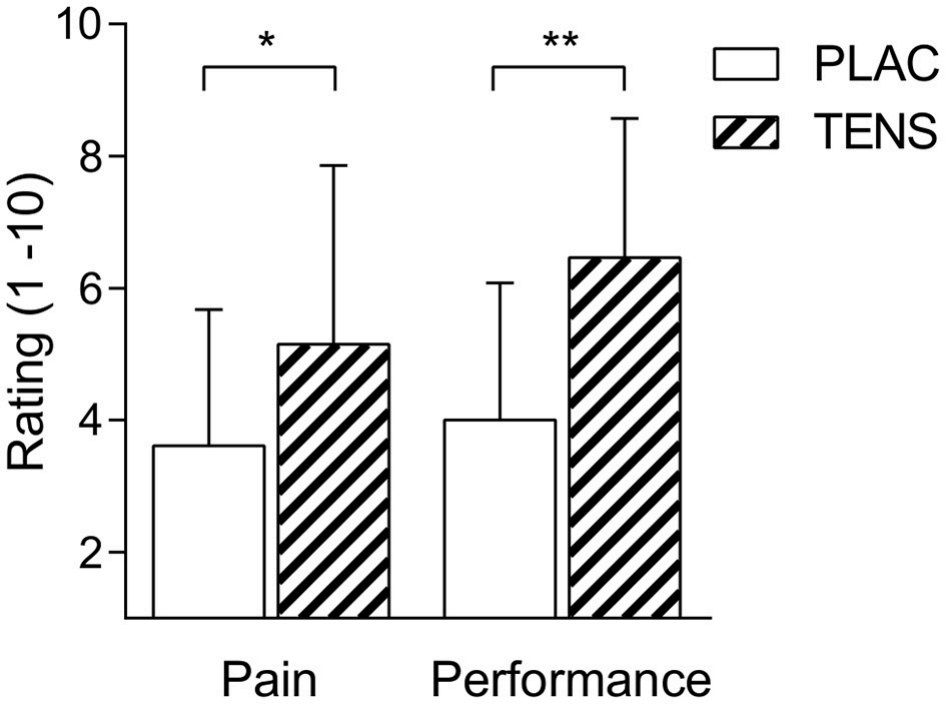
**Mean ± SD TENS belief ratings**. Participants ratings of pain relief (Pain) and subsequent influence on performance (Performance). PLAC, placebo; TENS, TENS condition. Ratings are expressed on a 1–10 scale (1; a bit, 10; a lot). ^*^ indicates a difference for pain, *P* < 0.05. ^**^ indicates a difference for performance *P* < 0.01.

There was a significant distance effect for RPE, FS and pain (*P* < 0.01), with all conditions having an increase in RPE and pain, whilst having a decrease in FS (Table 2). No significant interaction (Condition × distance) effect was found for any perceptual score, RPE (*P* = 0.58), FS (*P* = 0.68), and perceived quadriceps pain (*P* = 0.68) (Table 2). However, FS showed a moderate effect size and RPE had a large effect size for the interaction and trial effects. There was also no change in pressure pain thresholds attributed to TENS (see Tables 3, 4). Post study subjective ratings of the effectiveness of TENS were different between PLAC and TENS conditions for the level of pain relief [*t*_(12)_ = 2.68 *P* = 0.02, *d* = 1.55] and positive influence on performance [*t*_(12)_ = 4.68 *P* < 0.01, *d* = 2.70] (Figure 1) with large effect sizes.

**Table 2:** **Subjective ratings for exertion, affect and pain at each kilometer of TTs**.

	**1- km**	**2-km**	**3-km**	**4-km**	**5-km**	**Trial effect**	**Distance effect**	**Interaction**
**RPE**								
*CONT*	11.69 ± 1.44	12.85 ± 1.77	13.69 ± 1.93	14.88 ± 2.38	16.77 ± 1.96	*P* = 0.12 $\eta_{\text{p}}^{2}$ = 0.16	*P* < 0.01 $\eta_{\text{p}}^{2}$ = 0.82	*P* = 0.58 $\eta_{\text{p}}^{2}$ = 0.16
*PLAC*	11.85 ± 1.68	13.00 ± 1.41	14.31 ± 1.25	15.00 ± 1.58	17.31 ± 1.75			
*TENS*	12.23 ± 0.73	13.62 ± 1.12	14.54 ± 1.27	15.31 ± 1.65	17.15 ± 1.52			
**FS**								
*CONT*	1.69 ± 1.88	0.54 ± 1.71	−0.23 ± 1.92	−0.54 ± 2.15	−1.31 ± 2.46	*P* = 0.72 $\eta_{\text{p}}^{2}$ = 0.03	*P* < 0.01 $\eta_{\text{p}}^{2}$ = 0.69	*P* = 0.68 $\eta_{\text{p}}^{2}$ = 0.06
*PLAC*	1.15 ± 1.41	0.46 ± 1.56	−0.31 ± 1.84	−0.62 ± 2.14	−0.92 ± 2.84			
*TENS*	1.15 ± 1.57	0.38 ± 1.66	−0.31 ± 1.84	−0.85 ± 2.30	−1.31 ± 2.66			
**PAIN**								
*CONT*	2.19 ± 1.79	2.96 ± 2.09	3.92 ± 2.29	4.62 ± 2.73	6.12 ± 2.87	*P* = 0.68 $\eta_{\text{p}}^{2}$ = 0.03	*P* < 0.01 $\eta_{\text{p}}^{2}$ = 0.73	*P* = 0.68 $\eta_{\text{p}}^{2}$ = 0.05
*PLAC*	1.92 ± 1.31	2.92 ± 1.71	3.58 ± 1.89	4.38 ± 2.36	5.77 ± 2.62			
*TENS*	2.50 ± 2.21	2.81 ± 1.44	3.54 ± 1.90	4.35 ± 2.10	6.08 ± 2.36			

*Data presented as mean ± SD. RPE, Rate of perceived exertion; FS, feeling scale; Pain, perceived quadriceps pain; CONT, control. PLAC, placebo. TENS, TENS condition. $\eta_{p}^{\text{2}}$, partial eta squared. Significant distance effects are not shown*.

**Table 3:** **Raw changes in PPT, MVC, CAR, and Qtw,pot**.

	**BASE**	**PRE**	**POST**	**Trial effect**	**Time effect**	**Interaction**
**PPT (KGF)**						
*CONT*	5.23 ± 3.93	4.99 ± 3.57	5.35 ± 3.42	*P* = 0.47 $\eta_{\text{p}}^{2}$ = 0.07	*P* = 0.10 $\eta_{\text{p}}^{2}$ = 0.19	*P* = 0.90 $\eta_{\text{p}}^{2}$ = 0.02
*PLAC*	4.76 ± 3.28	4.53 ± 2.60	5.35 ± 4.30			
*TENS*	5.17 ± 2.32	5.12 ± 2.80	5.63 ± 3.82			
**MVC (N)**						
*CONT*	262.70 ± 78.04	254.19 ± 72.33	207.90 ± 63.19^*^	*P* = 0.97 $\eta_{\text{p}}^{2}$ < 0.01	*P* < 0.01 $\eta_{\text{p}}^{2}$ = 0.68	*P* < 0.05 $\eta_{\text{p}}^{2}$ = 0.18
*PLAC*	259.52 ± 84.58	251.71 ± 66.23	211.54 ± 66.23^*^			
*TENS*	271.16 ± 68.04	251.73 ± 74.32^#^	197.55 ± 61.96^*^			
**CAR**						
*CONT*	95.26 ± 3.16	95.18 ± 2.65	94.66 ± 4.36	*P* = 0.70 $\eta_{\text{p}}^{2}$ = 0.04	*P* = 0.81 $\eta_{\text{p}}^{2}$ = 0.02	*P* = 0.44 $\eta_{\text{p}}^{2}$ = 0.10
*PLAC*	94.41 ± 3.48	94.89 ± 2.25	95.46 ± 2.41			
*TENS*	96.72 ± 3.42	95.17 ± 2.93	95.19 ± 2.31			
**QTW,POT (N)**						
*CONT*	28.76 ± 8.75	26.93 ± 8.45	14.23 ± 7.15^*^	*P* = 0.10 $\eta_{\text{p}}^{2}$ = 0.17	*P* < 0.01 $\eta_{\text{p}}^{2}$ = 0.80	*P* = 0.22 $\eta_{\text{p}}^{2}$ = 0.11
*PLAC*	28.10 ± 7.84	27.44 ± 8.04	15.39 ± 8.44^*^			
*TENS*	30.34 ± 10.09	28.38 ± 9.82^#^	16.34 ± 9.87^*^			

Data presented as mean ± SD. BASE, baseline; PRE, pre-exercise; POST, post-exercise; PPT, pressure pain threshold; MVC, maximal voluntary contraction; CAR, Quadriceps central activation ratio, for CAR n = 10. Qtw,pot, potentiated quadriceps twitch. CONT, control; PLAC, placebo; TENS, TENS condition; $\eta_{p}^{\text{2}}$, partial eta squared; Time point effects

* *difference to BASE and PRE measurement*,

# *difference to BASE measurement*.

*Post-hoc tests for M-wave amplitude time effects revealed no difference between means*.

**Table 4:** **Percentage changes in PPT, MVC and CAR**.

	**BASE—PRE Exercise**	**PRE Exercise—POST Exercise**	**Trial effect**	**Time effect**	**Interaction**
**PPT (KGF)**					
*CONT*	−2.67 ± 19.07	6.65 ± 33.34	*P* = 0.98 $\eta_{\text{p}}^{2}$ < 0.01	*P* = 0.08 $\eta_{\text{p}}^{2}$ = 0.25	*P* = 0.99 $\eta_{\text{p}}^{2}$ < 0.01
*PLAC*	−1.71 ± 10.18	6.81 ± 22.49			
*TENS*	−2.56 ± 16.34	5.59 ± 22.37			
**MVC (N)**					
*CONT*	−2.51 ± 4.95	−17.46 ± 10.35^*^	*P* = 0.04 $\eta_{\text{p}}^{2}$ = 0.24	*P* < 0.01 $\eta_{\text{p}}^{2}$ = 0.66	*P* = 0.76 $\eta_{\text{p}}^{2}$ = 0.02
*PLAC*	−2.18 ± 7.05	−14.75 ± 12.89^*^			
*TENS*	−8.27 ± 8.92	−20.60 ± 13.74^*^			
**CAR**					
*CONT*	−0.06 ± 1.81	−0.54 ± 3.90	*P* = 0.38 $\eta_{\text{p}}^{2}$ = 0.10	*P* = 0.65 $\eta_{\text{p}}^{2}$ = 0.02	*P* = 0.50 $\eta_{\text{p}}^{2}$ = 0.07
*PLAC*	0.59 ± 3.13	0.65 ± 3.34			
*TENS*	−1.54 ± 3.20	0.07 ± 2.53			

Data presented as mean percentage change between measurements ± SD. BASE, baseline; PRE, pre-exercise; POST, post-exercise; PPT, pressure pain threshold; MVC, maximal voluntary contraction; CAR, Quadriceps central activation ratio, for CAR n = 10. CONT, control; PLAC, placebo; TENS, TENS condition. $\eta_{p}^{\text{2}}$, partial eta squared; Time point effects

* *difference to BASE—PRE. Post-hoc tests for MVC trial effect revealed no significant difference*.

### Overall performance

**Figure 2:**
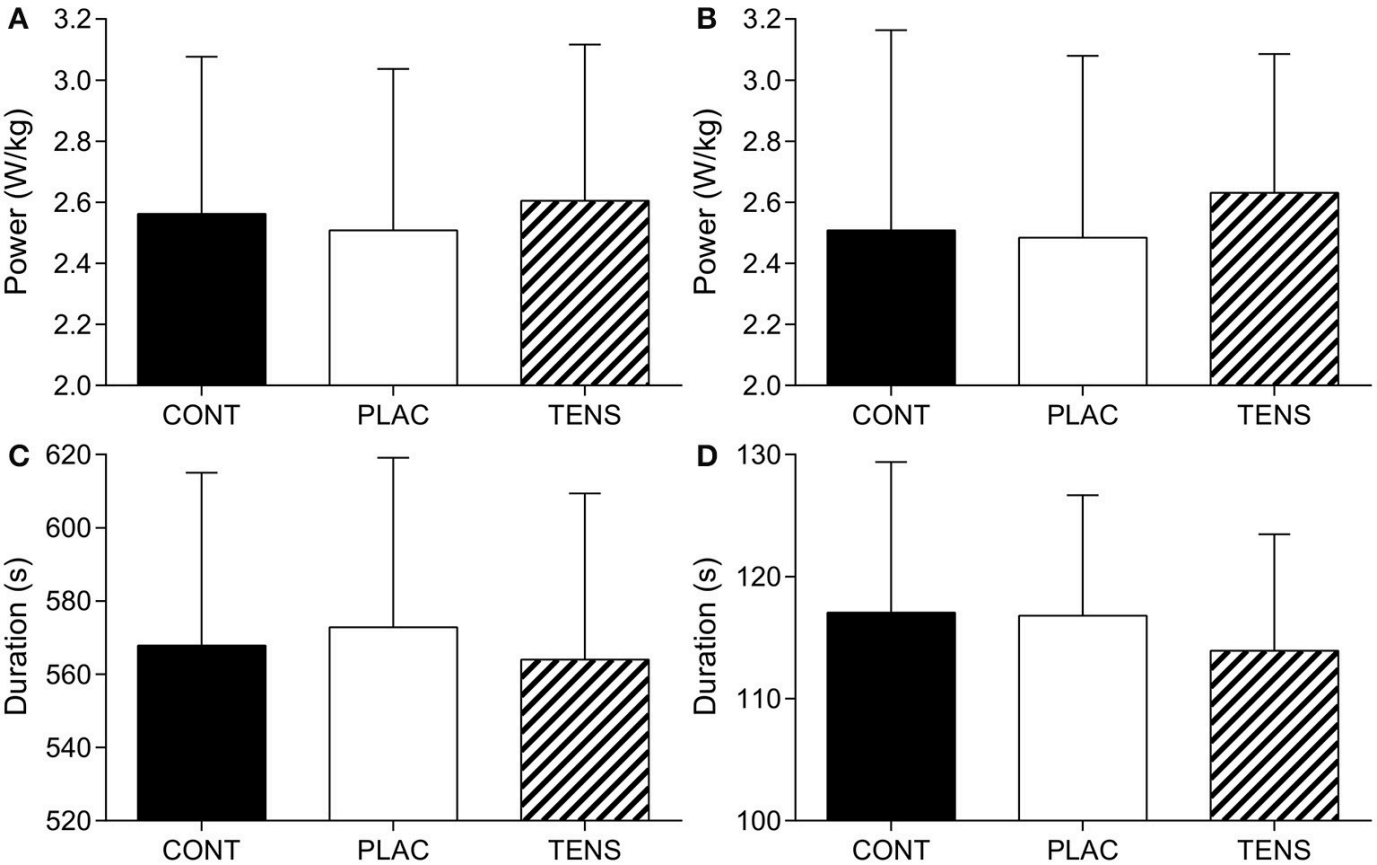
**Mean ± SD TT performance measures**. TT mean power output (W/kg) **(A)**, 1-km mean power output (W/kg) **(B)**, TT duration (s) **(C)**, and 1-km duration (s) **(D)**. CONT, control; PLAC, placebo; TENS, TENS condition.

**Figure 3:**
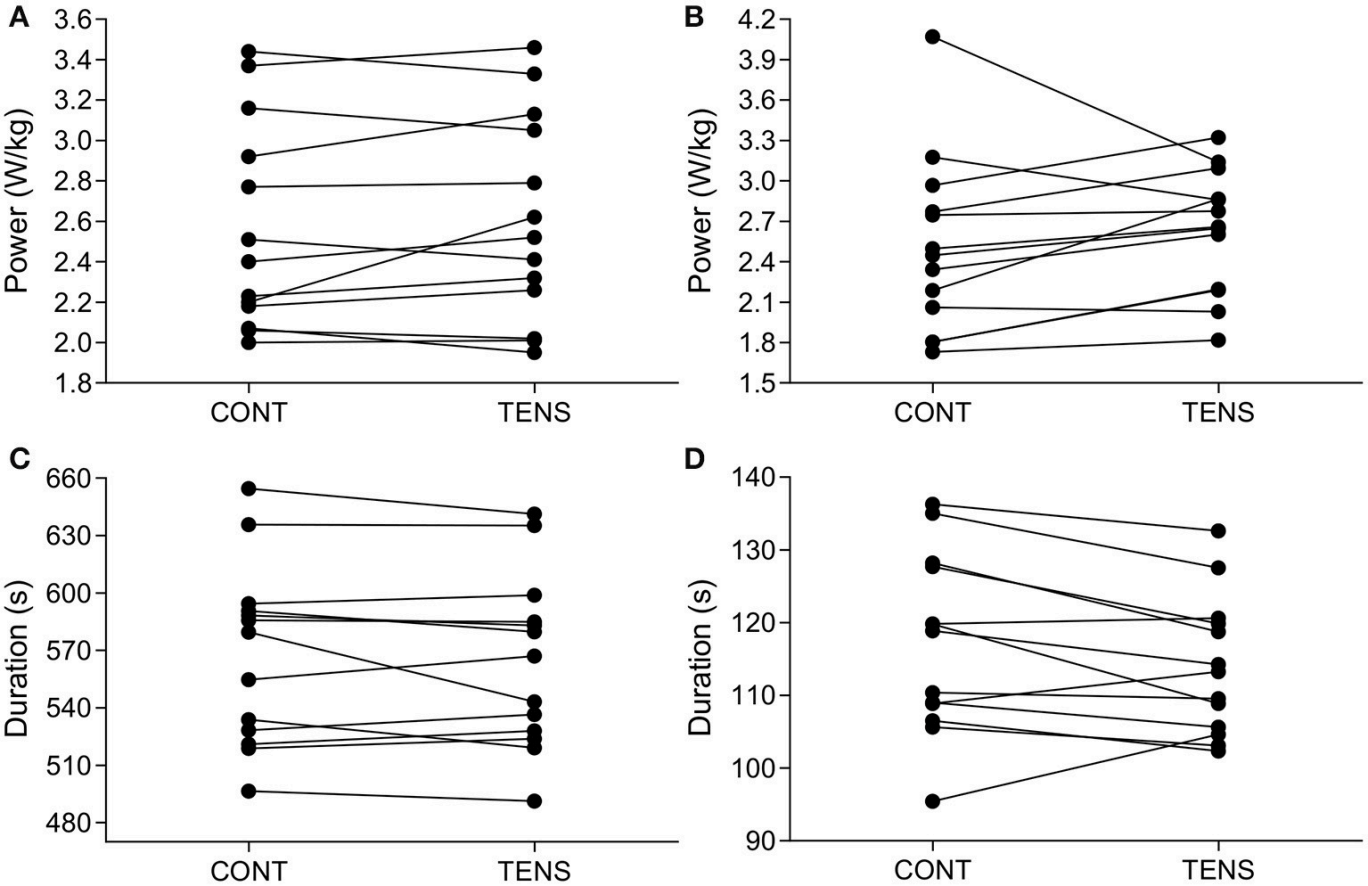
**Individual changes for TT performance measures**. TT mean power output (W/kg) **(A)**, 1-km mean power output (W/kg) **(B)**, TT duration (s) **(C)**, and 1-km duration (s) **(D)**. CONT, control; PLAC, placebo; TENS, TENS condition.

There was no significant difference for mean power maintained over the TT [*F*_(1.8, 21.8)_ = 2.0, *P* = 0.16, $\eta_{\text{p}}^{2}$ = 0.16], although there was a large effect size (Figure 2A). Completion time between conditions was not significantly different [*F*_(2, 24)_ = 1.9, *P* = 0.17, $\eta_{\text{p}}^{2}$ = 0.14] and had a large effect size (Figure 2C). Average power for the first kilometer was not significantly different [*F*_(1.4, 16.6)_ = 1.8, *P* = 0.21, $\eta_{\text{p}}^{2}$ = 0.13] (Figure 2B), as was duration [*F*_(1.8, 21.5)_ = 1.7, *P* = 0.20, $\eta_{\text{p}}^{2}$ = 0.13] (Figure 2D), however both had a moderate effect size. Visual inspection of data (Figure 2D) shows a decreased time in TENS condition. Of all participants, nine had a reduced time in the TENS condition at one kilometer compared to CONT (−3.12 ± 5.58 s to CONT, 95% CI; −6.49, 0.26) (Figure 3D). For total TT duration eight out of 13 improved their time in TENS compared to CONT (−3.82 ± 12.96 s to CONT, 95% CI; −11.65, 4.01) (Figure 3C).

### Pacing

**Figure 4:**
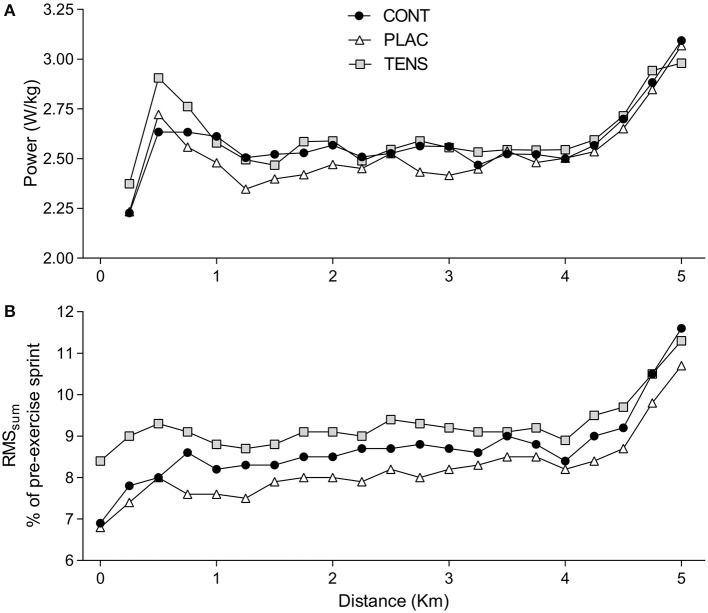
**TT pacing measures**. Group mean power output **(A)** and electromyography (RMSsum) profiles **(B)**. Mean power output is averaged over 250-m intervals. RMSsum data is reported as a percentage of a pre-exercise sprint value, RMSsum was measured for 20-s at 250-m intervals. Error bars have been excluded for clarity. CONT, control; PLAC, placebo; TENS, TENS condition.

There was no interaction (Condition × distance) effect for mean power over 250-m intervals [*F*_(38, 456)_ = 0.77, *P* = 0.83, $\eta_{\text{p}}^{2}$ = 0.06]. All TTs exhibited a similar pacing strategy, however there was greater variability in mean power in the first 750-m (Figure 4A). To investigate the variability at the start of the TTs, one-way repeated measures ANOVAs between distance points were conducted at 250-m [*F*_(1.78, 21.36)_ = 0.55 *P* = 0.56, $\eta_{\text{p}}^{2}$ = 0.04], 500-m [*F*_(1.66, 19.91)_ = 1.39 *P* = 0.27 $\eta_{\text{p}}^{2}$ = 0.10] and 750-m [*F*_(1.69, 20.33)_ = 1.31 *P* = 0.29, $\eta_{\text{p}}^{2}$ = 0.10]. Moderate effects sizes were observed at 500-m and 750-m. EMG data followed a similar pattern to power output with no significant interaction (Condition × distance) effect [*F*_(40, 480)_ = 1.01, *P* = 0.46, $\eta_{\text{p}}^{2}$ = 0.08] (Figure 4B). Furthermore, greater variability between TTs was shown with large effects at 250-m [*F*_(1.5, 18.3)_ = 2.31, *P* = 0.14, $\eta_{\text{p}}^{2}$ = 0.16], 500-m [*F*_(1.7, 20.3)_ = 2.05, *P* = 0.16, $\eta_{\text{p}}^{2}$ = 0.15] and 750-m [*F*_(1.6, 18.8)_ = 2.01, *P* = 0.17, $\eta_{\text{p}}^{2}$ = 0.14].

### Fatigue measurements

An exercise-induced reduction in MVC was recorded PRE to POST in all conditions (see Table 3), with only TENS application resulting in a significant reduction between BASE and PRE measurements. This difference was not apparent when expressed as a percentage change from BASE-PRE measurements (see Table 4). Rather, MVC revealed a main effect of time with an exercise-induced reduction in all conditions, but no significant interaction (Condition × time). Mean Qtw,pot followed MVC values with all conditions having an exercise-induced reduction PRE-POST (see Tables 3, 5). Only TENS condition had a reduction in raw Qtw,pot between BASE and PRE measurements, but a non-significant percentage change (BASE-PRE) (see Table 5). There was no reduction in quadriceps CAR as a raw value (see Table 3) or as a percentage change (see Table 4). Percentage change responses to magnetic stimulation are shown in Table 5. There was an exercise-induced reduction in all measures, except M-wave variables.

**Table 5:** **Percentage changes in evoked responses to magnetic stimulation**.

	**BASE—PRE Exercise**	**PRE Exercise—POST Exercise**	**Trial effect**	**Time effect**	**Interaction**
**QTW,POT (N)**					
*CONT*	−5.09 ± 13.11	−46.75 ± 17.59^*^	*P* = 0.48 $\eta_{\text{p}}^{2}$ = 0.06	*P* < 0.01 $\eta_{\text{p}}^{2}$ = 0.83	*P* = 0.38 $\eta_{\text{p}}^{2}$ = 0.08
*PLAC*	−2.25 ± 8.02	−44.56 ± 20.52^*^			
*TENS*	−5.92 ± 9.11	−41.43 ± 26.33^*^			
**MRFD (N.S^−1^)**					
*CONT*	−3.91 ± 11.92	−56.73 ± 19.73^*^	*P* = 0.48 $\eta_{\text{p}}^{2}$ = 0.06	*P* < 0.01 $\eta_{\text{p}}^{2}$ = 0.86	*P* = 0.24 $\eta_{\text{p}}^{2}$ = 0.11
*PLAC*	−2.62 ± 9.31	−54.14 ± 20.78^*^			
*TENS*	−3.35 ± 16.81	−48.54 ± 23.32^*^			
**CT (S)**					
*CONT*	−0.61 ± 12.50	−23.78 ± 13.92^*^	*P* = 0.44 $\eta_{\text{p}}^{2}$ = 0.07	*P* < 0.01 $\eta_{\text{p}}^{2}$ = 0.65	*P* = 0.33 $\eta_{\text{p}}^{2}$ = 0.09
*PLAC*	−1.77 ± 18.50	−20.24 ± 12.60^*^			
*TENS*	4.74 ± 12.30	−23.87 ± 11.37^*^			
**MRR (N.S^−1^)**					
*CONT*	−6.03 ± 19.81	−59.36 ± 15.22^*^	*P* = 0.22 $\eta_{\text{p}}^{2}$ = 0.12	*P* < 0.01 $\eta_{\text{p}}^{2}$ = 0.88	*P* = 0.78 $\eta_{\text{p}}^{2}$ = 0.01
*PLAC*	−2.70 ± 23.36	−53.31 ± 22.34^*^			
*TENS*	−0.40 ± 10.04	−55.59 ± 23.30^*^			
**RT_0.5_ (N.S^−1^)**					
*CONT*	−5.09 ± 13.25	−23.32 ± 19.02^*^	*P* = 0.19 $\eta_{\text{p}}^{2}$ = 0.13	*P* < 0.01 $\eta_{\text{p}}^{2}$ = 0.56	*P* = 0.12 $\eta_{\text{p}}^{2}$ = 0.16
*PLAC*	−3.15 ± 23.57	−21.98 ± 18.74^*^			
*TENS*	7.09 ± 17.06	−26.85 ± 15.85^*^			
**M-WAVE AMPLITUDE (MV)**					
*CONT*	2.63 ± 18.83	4.75 ± 13.50	*P* = 0.22 $\eta_{\text{p}}^{2}$ = 0.12	*P* = 0.07 $\eta_{\text{p}}^{2}$ = 0.26	*P* = 0.45 $\eta_{\text{p}}^{2}$ = 0.06
*PLAC*	−0.66 ± 7.73	3.03 ± 10.91			
*TENS*	1.89 ± 16.14	13.85 ± 21.50			
**M-WAVE DURATION (MS)**					
*CONT*	−4.71 ± 14.21	−7.11 ± 24.17	*P* = 0.17 $\eta_{\text{p}}^{2}$ = 0.14	*P* = 0.49 $\eta_{\text{p}}^{2}$ = 0.04	*P* = 0.25 $\eta_{\text{p}}^{2}$ = 0.11
*PLAC*	1.07 ± 17.25	7.96 ± 16.49			
*TENS*	−9.07 ± 37.90	−7.05 ± 27.86			

Data presented as mean percentage change between measurements ± SD. BASE, baseline; PRE, pre-exercise; POST, post-exercise; Qtw,pot, potentiated quadriceps twitch; MRFD, maximal rate of force development; CT, contraction time; MRR, maximal rate of relaxation; RT_0.5_, one-half relaxation time; CONT, control; PLAC, placebo; TENS, TENS condition; $\eta_{p}^{\text{2}}$, partial eta squared. Time point effects

* *difference to BASE—PRE*.

## Discussion

This is the first study to investigate the influence of TENS administered prior to a 5-km TT, on exercise-induced pain, with a focus on influencing exercise intensity and increasing performance. It was hypothesized, TENS application would adjust the threshold for sensing pain, allowing for increased power output for the same rating of pain. As hypothesized, TENS failed to significantly influence within-exercise subjective pain ratings, but no significant effect on pacing and performance was observed. However, a large effect size for TT duration and mean power indicate a possible difference in favor of TENS compared with PLAC. At the start of trials, moderate to large effect sizes indicate differences in power output and EMG data. This suggests a possible influence of TENS on anticipation and, consequently, the selection of an initial exercise intensity.

### Pacing and performance

The application of TENS was associated with a large effect on EMG and a moderate effect on power output at the start of the TT, suggesting that TENS application may have influenced participant's anticipation of the task (see Figures 2–4). Afferent feedback is important in setting an initial exercise intensity (Tucker, 2009). This is evident with reduced starting exercise intensities when homeostasis is threatened (Amann et al., 2007; Schlader et al., 2011). Consequently, exercise intensity is reduced in order to limit excessive levels of fatigue and maintain homeostasis. Our results seem to indicate that TENS application possibly limits afferent feedback activity prior to exercise, resulting in greater muscle recruitment (as inferred from EMG data) and power output at the start of the TT. This supports research indicating a higher intensity is chosen when afferent information is removed or modified (Amann et al., 2009). It is likely that this occurs due to the lack of feedback to inform on potential or actual muscle damage and metabolic activity. This would indicate to the brain that more work can be done without indication of serious consequences, creating a greater neural drive to exercising muscle.

TENS is shown to influence motor neuron excitability (Hopkins et al., 2002; Pietrosimone et al., 2009). In this perspective, it is interesting to note that TENS increased EMG activity, a surrogate for muscle recruitment (Ansley et al., 2004), at the start of the TT. This enhanced central neural drive was concomitant to a higher power output. Importantly, TENS did not produce any change in M-wave responses from BASE to PRE, indicating no change in resting muscle function before the cycling TT (see Table 5). However, another possible explanation for this result is that neuromuscular electrical stimulation can change voluntary muscle recruitment patterns, allowing for non-sequential activation of muscle fibers (Gregory and Bickel, 2005). Yet, stimulation protocols showing these effects are different to the method utilized in this study. It is possible this effect occurred, creating a poor recruitment of muscle after TENS stimulation which may require a greater neural drive to be produced. Alternatively, there is an association between TENS and greater local blood flow (Hallen et al., 2010). This may lead to a greater activation of type I muscle fibers which are related to cycling efficiency, thus increasing EMG activity (Coyle et al., 1992). These possible factors may explain the observed increase in EMG readings, allowing for higher muscle recruitment and greater power output during the early part of the TENS condition TT. However, we cannot identify the primary source from this investigation.

After the initial differences, all TTs exhibit a similar power output and EMG readings beyond one kilometer (Figure 4). An initial aggressive pacing strategy would likely assist performance in shorter tasks such as a 5-km TT (Abbiss and Laursen, 2008). This would create greater mechanical and chemical stimuli likely to trigger conscious awareness and influence pain perceptions (Swart et al., 2012; Edwards and Polman, 2013; Mauger, 2014). It is possible that this occurred in our study at approximately one kilometer, with increased exercise-induced stimuli diminishing the effectiveness of TENS. This may indicate that afferent information from active skeletal muscles is now unaffected and the participant uses this to pace performance. It is also likely at this stage of the intense exercise, feedback from a number of different sources, not just the active muscle, is pushing the individual close to their tolerance limit (Hureau et al., 2016). For these reasons, even with the non-significant differences in intensity at the start of the trial, it is not surprising observe similar subjective responses for pain, feeling and exertion in all conditions. Therefore, these results support the theory that exercise is regulated in part by afferent feedback to a perceived pain threshold (Mauger et al., 2010), which presumably plays a role in a global sensory tolerance limit (Hureau et al., 2016).

### Practical implications

Application of TENS did not provide any overall performance improvement for a 5-km TT. However, there was a large effect size for TT duration and moderate effect size for duration of the first kilometer (see Figures 2, 3). These results provide some support for the potential of TENS to increase exercise performance. Participants completed the first kilometer of the TTs within a range of 95.44–136.28-s, and the difference between TENS and CONT conditions being −3.12 ± 5.58-s. Therefore, this research identifies that any possible benefit of TENS administered prior to exercise may be limited to events of ≤ 2 min and where exercise-induced pain is localized. However, the possible reduction in pain to increase exercise intensity poses ethical concerns for athlete safety. Administering an analgesic intervention will augment stimuli that warns of potential muscle damage, this creates the potential for a greater risk of injury through increased exercise intensity. Our study found no significant impact on exercise intensity, but also no indication of greater exercise-induced fatigue due to TENS application (i.e., PRE-POST measurement, see Tables 3–5). It may be of benefit for future investigations to confirm if effects of TENS application occur in elite populations, to highlight any potential benefits or concerns of TENS use.

One possible future investigation could look at potential benefits of TENS use within a task. We tested the use of TENS prior a task, with a 5-km TT chosen in anticipation that exercise would be conducted in a proposed post-stimulation analgesic period. Compared to a longer task (e.g., 20-km TT), a greater exercise intensity would be observed in a 5-km TT, and therefore, a greater nociceptive stimulus is expected. Accordingly, when pain is greater, it may be harder to distinguish small changes in pain perceptions that an intervention may provide. Also theoretically, it is possible that TENS could be beneficial for longer duration tasks which are more reliant on afferent feedback for regulation (Tucker, 2009; Mauger et al., 2010), and where pain perceptions are expected to be less prominent. Furthermore, TENS is more likely to reduce pain perceptions when stimulation is active. Therefore, future investigations into the possible use of TENS to enhance exercise performance may look at utilizing TENS during trials of greater length. Ethically, it is unlikely TENS could be used within a sporting event due to doping concerns, but there may be merit in use of TENS as a within-exercise training intervention (Hughes et al., 2013).

### Limitations

There are several limitations in this study. Perceptions of TENS for pain relief and influence on performance were greater than PLAC condition (see Figure 1). Measures were taken to minimize the influence of any placebo effect of TENS. However, participants would have clearly felt a difference in sensation between TENS and PLAC conditions. Furthermore, participants were made aware of the aims of the study, and informed they were receiving high or low TENS, but not aware of which intervention was placebo. This could have had implications on the results shown by TENS (Son et al., 2016). Placebo effects have been shown to influence exercise pain perceptions (Benedetti et al., 2007) but also the ability to produce force (Broatch et al., 2014). As participant's perceptions of TENS effectiveness on pain relief and performance were increased post study compared to PLAC condition (Figure 1), this could have produced changes at the start of the 5-km TT.

With physiological differences between participants, it is possible that there are responders and non-responders to this type of intervention (Figure 3). For example, the amount of subcutaneous fat may affect the amplitude of stimulation to afferent fibers (Hughes et al., 2013). Hence, those with lower body fat may not be able to increase the amplitude of stimulation to a level that will stimulate deeper tissue (Hughes et al., 2013). This could possibly result in different levels of stimulation between participants, leading to differing levels of afferent information, and effect on performance. This is a limitation of the study, as the final current intensity was not recorded, we cannot confirm the dose received by participants. However, application of TENS was adjusted to an individual's own sensory threshold, in effect an individual's tolerance of the stimulation. It has to be noted that applying a TENS intensity higher than what a participant can tolerate would be unethical. However, it could be speculated that those who responded to the intervention (i.e., higher initial power output) (Figures 2, 3) were able to tolerate a higher current intensity during TENS application.

The potential of TENS to affect exercise-induced pain was based on previous research in pain-free individuals that influenced PPT (Moran et al., 2011), but also restored muscle strength when pain was induced (Son et al., 2016). Recent research however, has indicated a possibility for different subgroups of group III and IV muscle afferents which are sensitive to distinct metabolites. One subgroup is likely to respond to intramuscular metabolites associated with aerobic exercise, whilst another responds to noxious levels of metabolites (e.g., hypertonic saline) associated with ischemic contractions (Amann et al., 2015). These differing characteristics of muscle afferents may be a possible reason why TENS failed to significantly change exercise intensity within this study. Whilst in comparison, analgesic effects are demonstrated during exercise when pain is induced (Son et al., 2016).

Reductions in MVC and Qtw,pot were observed from BASE to PRE for TENS condition (see Table 3; Moran et al., 2011; Amann et al., 2015; Son et al., 2016), although percentage reductions were not significantly different (see Tables 3, 4). This likely resulted from the stimulation intensity being close to the threshold for muscle contraction. However, despite this apparent fatigue, it dissipated quickly as power was greatest in TENS condition early in the TT (see Figure 4A). Therefore, a possible limitation of prior to exercise use of TENS may be the application settings. Application duration should be limited and higher intensities avoided as this may induce peripheral fatigue that is detrimental to performance. Furthermore, PRE assessment was conducted after the cycling warm-up, which may have contributed to the reduced voluntary force, thus disguising the true influence of TENS on muscle strength properties. Settings for TENS were based on previous research indicating this would activate the gate control of pain and reduce feedback from group III and IV afferents (Moran et al., 2011). However, amplitude, stimulation duration, prior use and tolerance of opioids can compromise TENS effectiveness (Sluka et al., 2013). Participants currently taking pain medication were excluded from the study, but the prior use of opioids was not recorded. Typically, peak effects for analgesia provided by TENS will be greater when stimulation is active, or immediately after cessation (Moran et al., 2011; Vance et al., 2012). For this research, we conducted a 5-km TT, as we anticipated post-stimulation effects for approximately 30-min would be present (Moran et al., 2011). As expected, within-exercise pain perceptions were similar, but there is an absence of significant differences in exercise intensity. Therefore, by administering the intervention prior to exercise, it is possible that analgesic effectiveness of TENS for this mode of exercise is reduced.

Although the current study has several limitations, the overall multidisciplinary approach allows for assessment of a number of variables related to exercise performance. The aim of the study was to assess the efficacy of TENS on exercise performance, with a focus on exercise-induced pain. Although there were no significant differences in pain and performance, the reporting of multiple variables related to exercise intensity, fatigue but also psychological aspects represent strengths of this study.

### Conclusions

In conclusion, this study found no significant effects of TENS administered prior to exercise on 5-km TT performance, although similar pain perceptions were observed. This casts doubt on the effectiveness of the application of TENS prior to exercise to modify afferent feedback and influence perceptions of exercise-induced pain. However, there are indications TENS application may influence neural drive and power output at the start of a 5-km TT. Aside from any potential changes in pain perceptions, other possible reasons for this include psychological belief in the intervention and altered muscle recruitment. Future research could investigate the effectiveness of TENS on modifying the sensation of exercise-induced muscle pain, with a focus on application during other forms of aerobic exercise.

## Author contributions

All authors contributed to the study design. AH collected data, with AH, FB and RP conducted the initial data analysis. First draft of manuscript was written by AH, with all authors reviewing the manuscript and approving the final version.

### Conflict of interest statement

The authors declare that the research was conducted in the absence of any commercial or financial relationships that could be construed as a potential conflict of interest.
